# Selective removal of magnesium from lithium‐rich brine for lithium purification by synergic solvent extraction using β‐diketones and Cyanex 923

**DOI:** 10.1002/aic.16246

**Published:** 2020-04-21

**Authors:** Zheng Li, Koen Binnemans

**Affiliations:** ^1^ Department of Chemistry KU Leuven Heverlee Flemish Brabant Belgium

**Keywords:** Cyanex 923, β‐diketone, lithium, magnesium, synergic solvent extraction

## Abstract

In the production of battery‐grade and high‐purity Li_2_CO_3_, it is essential to remove magnesium impurities. The state‐of‐the‐art solvent extraction (SX) process using Versatic Acid 10 and D2EHPA co‐extracts 3.3–5.5% lithium, while removing 86–98% magnesium. Here, we demonstrate that synergic SX systems containing a β‐diketone (HPMBP, HTTA or HDBM) and Cyanex 923 are highly selective for magnesium extraction over lithium (separation factor *α* > 1,000). The extracted magnesium and lithium complexes have the stoichiometry of [Mg∙A_2_∙(C923)_2_] and [Li∙A_*x*_∙(C923)_2_] (*x* = 1, 2), respectively (A represents deprotonated β‐diketone). The three β‐diketone synergic SX systems all considerably outperformed the Versatic Acid 10 system for magnesium removal from a synthetic solution containing 24 g L^−1^ Li and 0.24 g L^−1^ Mg. In a three‐stage batch counter‐current extraction, the HPMBP and Cyanex 923 synergic SX system removed 100% magnesium with only 0.6% co‐extraction of lithium. This excellent Mg/Li separation is the best result reported so far.

## 1 INTRODUCTION

Powering vehicles by lithium‐ion‐batteries (LIBs) instead of fossil fuels is one of the important solutions to counteract global warming by reducing CO_2_ emissions.^1^, ^2^ Driven by the increasing popularity of electric vehicles, not only has the share of lithium (Li) used in batteries increased to 56% of the global Li consumption in 2018,^3^ the global consumption of Li has also increased significantly in the past 10 years.^3^, ^4^, ^5^ In the coming three decades, the global Li demand is expected to continuously increase: it will triple by 2025 compared to that in 2015^6^, ^7^; and it will be 27 times that of 2012 when 20% of the vehicle fleet is expected to be fully electric vehicles powered by LIBs in 2050.^8^, ^9^

Li_2_CO_3_ is a key precursor of LIBs. It is produced from two main sources: pegmatites and brines, with the latter comprising over 70% of the global exploitable Li resources.^10^ Currently, the majority of the global Li_2_CO_3_ is produced from brines.^11^, ^12^ Magnesium (Mg) always accompanies Li in salt lake brines, consequently Mg impurities are often found in Li_2_CO_3_. The typical process for the production of Li_2_CO_3_ from brines includes: (a) concentration of brines by evaporation in a solar pond for about 1 year, (b) removal of Mg by lime milk (Ca[OH]_2_) and (c) removal of calcium by addition of Na_2_CO_3_/Li_2_CO_3_, and finally (d) precipitation of Li_2_CO_3_ by addition of Na_2_CO_3_.^13^, ^14^, ^15^ Li_2_CO_3_ produced from this process is of technical‐grade (~99%), but it is difficult to reach battery‐grade (>99.5%) and high‐purity (99.99%) Li_2_CO_3_.^16^ High‐purity (and battery‐grade) Li_2_CO_3_ is usually further processed from technical‐grade Li_2_CO_3_ by re‐dissolution and further purification (such as by ion‐exchange).^17^ A more efficient and direct way to produce high‐purity (and battery‐grade) Li_2_CO_3_ would be the removal of the residual Mg (and Ca) from concentrated brine before precipitation of Li_2_CO_3_. However, this route is also challenging because Mg and Li exhibit similar chemical properties due to their diagonal relationship in the periodic table.

Solvent extraction (SX) is perhaps the most widely used technique for the separation and purification of metals ions and it is promising for selective removal of residual Mg from Li‐rich brine. There are several studies on the extraction of Mg from Li‐containing solutions. Bukowsky et al. studied the extraction of Ca and Mg using di‐(2‐ethylhexyl)phosphoric acid (D2EHPA) from a feed solution containing about 6.9 g L^−1^ Li, 4.0 g L^−1^ Ca and 0.49 g L^−1^ Mg.^18^ Under the optimized condition, >98% Ca and >98% Mg was removed, with co‐extraction of 3.4% Li. Zhang et al. studied the extraction of Mg from a synthetic brine solution (19.5 g L^−1^ Mg and 0.021 g L^−1^ Li) for the separation of Mg and Li using saponified D2EHPA, but the co‐extraction of Li was quite significant.^19^ Virolainen et al. compared the performance of D2EHPA and Versatic Acid 10 (a mixture of branched carboxylic acids with the common structural formula of C_10_H_20_O_2_) for the removal of Ca and Mg from a synthetic concentrated brine (26.0–34.0 g L^−1^ Li, 1.14–1.55 g L^−1^ Ca and 0.045–0.075 g L^−1^ Mg) for purification of Li.^16^ It was found D2EHPA and Versatic Acid 10 overall performed similarly, but D2EHPA had a higher metal loading capacity, while Versatic Acid 10 had a better Mg/Li selectivity. Under the optimized conditions of a two‐stage process, > 98% Ca and 86–98% Mg were removed with co‐extraction of 3.3–5.5% Li. Recently, Binnemans et al. developed a process using a binary extractant, [A336][V10], for removal of Mg from a synthetic brine solution (15 g L^−1^ Mg and 0.20 g L^−1^ Li) for Li recovery.^20^ The process could remove >98% Mg, while co‐extracting about 10% Li. In summary, D2EHPA and Versatic Acid 10 could selectively remove Mg from Li, but the remaining Mg in the raffinate is still considerable and the co‐extraction of Li is also quite significant.

**FIGURE 1:**
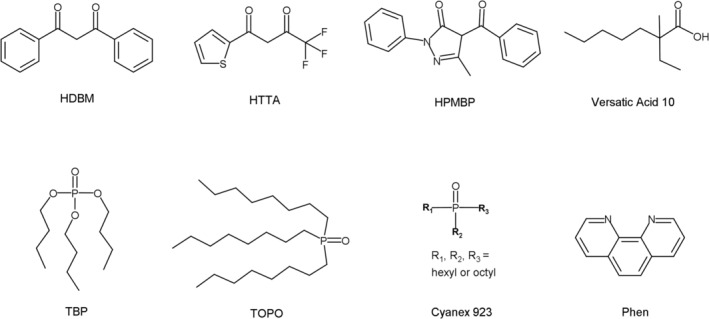
Structures of extractants for Li extraction: dibenzoylmethane (HDBM), 2‐thenoyltrifluoroacetone (HTTA), 4‐benzoyl‐3‐methyl‐1‐phenyl‐2‐pyrazolin‐5‐one (HPMBP), Versatic Acid 10 (a mixture of branched carboxylic acids with the common structural formula of C_10_H_20_O_2_, one isomer is shown), tri‐*n*‐butyl phosphate (TBP), tri‐*n*‐octyl phosphine oxide (TOPO), Cyanex 923 (a mixture of trialkyl phosphine oxides mainly with *n*‐hexyl and *n*‐octyl groups), and 1,10‐phenanthroline (Phen)

It seems that the Mg/Li selectivity of single‐extractant SX systems (Such as D2EHPA and Versatic Acid 10) is limited. Synergic SX systems (using mixtures of two or more extractants) often have much‐improved separation performance compared with single‐extractant systems.^21^, ^22^, ^23^, ^24^ β‐diketones and some neutral ligands have been found to have synergism for Li extraction since the 1960s. Various β‐diketones, including dibenzoylmethane (HDBM), 2‐thenoyltrifluoroacetone (HTTA), benzoyltrifluoroacetone (HBTA), 1‐phenyl‐1,3‐isodecanedione (LIX 54), 4,4,4‐trifluoro‐1‐(4‐tetrapropylenephenyl)‐1,3‐butanedione (LIX 51), and so on, have been investigated. While the neutral ligands studied include tri‐*n*‐butyl phosphate (TBP), tri‐*n*‐octyl phosphine oxide (TOPO), Cyanex 923 (a mixture of trialkyl phosphine oxides mainly with *n*‐hexyl and *n*‐octyl groups) and 1,10‐phenanthroline and its derivatives.^25^, ^26^, ^27^, ^28^, ^29^, ^30^, ^31^ The structures of the extractants are shown in Figure 1. Besides the normal β‐diketones, 4‐acyl‐5‐pyrazolones are also a type of β‐diketones and they can also extract Li in combination with neutral ligands but the working pH is lower due to their lower pKa values.^32^, ^33^, ^34^, ^35^ Among these studies, only Umetani et al. investigated the extraction of Mg using 1‐phenyl‐3‐methyl‐4‐acylpyrazol‐5‐ones,^32^ all other studies mainly focused on the separation of Li from Na and K.

In this study, we describe the performance of β‐diketone and neutral ligand synergic SX systems for the selective removal of Mg for purification of Li that is essential for the production of batter‐grade and high‐purity Li_2_CO_3_. Among the neutral ligands investigated, 1,10‐phenanthroline and its derivatives are expensive and have high solubility in water, and TBP is inferior to TOPO and Cyanex 923 for synergism. Comparing TOPO and Cyanex 923, two similar compounds, the latter is a liquid at room temperature and is readily soluble in various diluents, hence Cyanex 923 was selected as the best candidate. Among the β‐diketones, HDBM (pK_a_ = 9.68^36^), HTTA (pK_a_ = 6.25^37^), and 4‐Benzoyl‐3‐methyl‐1‐phenyl‐2‐pyrazolin‐5‐one (HPMBP, pK_a_ = 3.92^38^) were chosen for their varying pK_a_ values. In addition to extraction performance, we illustrate the mechanism of Mg and Li extraction by the synergic SX system and develop a process for Mg removal.

## 2 EXPERIMENTAL SECTION

### 2.1 Chemicals

Cyanex® 923 (90%) was obtained from Cytec Industries B.V. (Vlaardingen, Netherlands); Versatic Acid® 10 (>90%) was obtained from Resolution Europe B.V. (Amsterdam, Netherlands); NaOH (analytical reagent) and HCl (37%) were supplied by Fisher Scientific (Merelbeke, Belgium); LiCl (≥99.5%) was obtained from Carl Roth (Karlsruhe, Germany); MgCl_2_ (99%), LiOH (98%), tris(hydroxymethyl)aminomethane (Tris buffer, ACS reagent), dibenzoylmethane (98%) and *p*‐cymene (>99%) were purchased from Acros Organics (Geel, Belgium); 4‐benzoyl‐3‐methyl‐1‐phenyl‐2‐pyrazolin‐5‐one (>98%) was purchased from Alfa Aesar (Karlsruhe, Germany); chloroform‐*d* (99.8%), D_2_O (99.9%) and 2‐(*N*‐morpholino)ethanesulfonic acid (MES buffer, 99.5%) were obtained from Sigma−Aldrich (Diegem, Belgium); Li and Mg standard solutions (1,000 ± 10 mg L^−1^) and sodium dihydrogen phosphate (>98%) were purchased from Chem‐Lab (Zedelgem, Belgium); acetic acid (100%) was purchased from VWR Chemicals (Leuven, Belgium); 2‐thenoyltrifluoroacetone (98%) was purchased from Fluorochem (Hadfield, UK). Milli‐Q water (18.2 MΩ cm at 298.2 K) was used to prepare the aqueous solutions. All chemicals were used as received, without any further purification.

### 2.2 Experimental procedures

#### 2.2.1 Extraction experiments

The aqueous solutions were buffered using appropriate buffer solutions to maintain the desired pH when the effect of pH needed to be considered. Each extraction experiment was carried out in a 15 ml centrifuge tube with 5.0 ml of the aqueous solution and 5.0 ml of the organic solution containing the extractants dissolved in *p*‐cymene. *p*‐Cymene (flash point 47°C) is a solvent that can be derived from bio‐mass and can be a greener substitute of toluene for dissolving aromatic compounds.^39^ It can be regarded as a model of the commercial diluent SOLVESSO 150 Fluid (flash point 66°C), which is dedicated to solvent extraction applications. Mixtures of the two phases were shaken for 30 min at 300 rpm using a Thermo Scientific 2000 shaker to attain extraction equilibrium. Afterward, the samples were centrifuged for 3 min at 4000 rpm in a Heraeus Megafuge 1.0 centrifuge to accelerate phase separation. Stripping was carried out following the same method as extraction using 1.0 ml of 0.50 mol L^−1^ HCl and 1.0 ml of the loaded organic phase. Organic‐to‐aqueous phase ratios (O/A) were varied by using 1.0, 2.0, 3.0, or 5.0 ml aqueous solutions while varying the organic phase accordingly. The aqueous phases at equilibrium and the resultant aqueous solutions after stripping were analyzed for Mg and Li concentrations by inductively coupled plasma optical emission spectroscopy (ICP‐OES).

The percentage extraction %*E*, the distribution ratio *D* and the separation factor *α* are defined as:

(1) $$\%E=\frac{c_{\mathrm{org}}∙V_{\mathrm{org}}}{c_{\mathrm{org}}∙V_{\mathrm{org}}+c_{\mathrm{aq}}∙V_{\mathrm{aq}}}\times 100\%$$

(2) $$D=\frac{c_{\mathrm{org}}}{\;c_{\mathrm{aq}}}$$

(3) $$\alpha =\frac{D_{A}}{\;D_{B}}$$

where *c*_org_ and *c*_aq_, *V*_org_ and *V*_aq_ are concentrations and volumes of the organic and the aqueous phase at extraction equilibrium, respectively; *D*_A_ and *D*_B_ are the distribution ratios of metals A and B, respectively.

The synergism factor *R* is defined as:

(4) $$R=\frac{D^{\mathrm{mix}}}{D^{L1}+D^{L2}}$$

where *D* ^L1^, *D* ^L2^ and *D* ^mix^ are the distribution ratios of a metal ion when extracted by the extractant L_1_ and L_2_ alone and by a mixture of the two. *R* > 1 means that the mixture has synergism, and *R* < 1 means antagonism.

#### 2.2.2 Batch counter‐current extraction test

A three‐stage batch counter‐current extraction was conducted to simulate a continuous multistage counter‐current solvent extraction. The flow sheet can be found in Figure 13. Up to 8 extraction cycles (5.0 ml of the aqueous phase and 5.0 ml of the organic phase in each extraction) were carried out to ensure a steady state in the extraction process. Each batch was shaken for 10 min (5 min is sufficient to reach equilibrium, Figure S1). The concentration of Mg and Li in all the loaded organic phases, all the raffinates and the two phases of the last three batches were determined.

#### 2.2.3 Analytical instruments

Mg and Li were analyzed using a Perkin Elmer Optima 8300 ICP‐OES equipped with a Scott Cross‐Flow nebulizer. A Bruker AVANCE NEO 400 and a Bruker AVANCE NEO 600 nuclear magnetic resonance spectroscopy device was used to record the ^1^H NMR (400 MHz) spectra and ^7^Li NMR spectra (233 MHz). pH was measured by a Mettler‐Toledo SevenCompact pH meter in combination with a Mettler‐Toledo InLab Micro glass electrode.

## 3 RESULTS AND DISCUSSIONS

### 3.1 Synergism between β‐diketone and Cyanex 923

**FIGURE 2:**
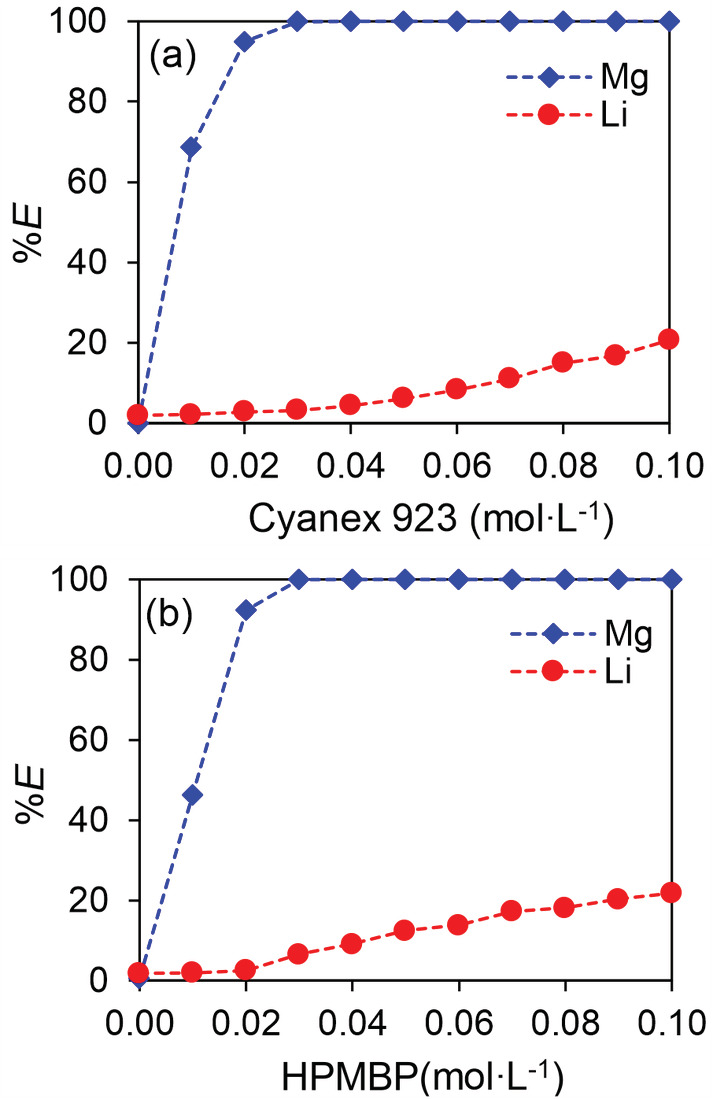
Synergism of Mg and Li extraction by HPMBP and Cyanex 923. (a) 0.10 mol L^−1^ HPMBP and varying Cyanex 923 concentrations; (b) 0.10 mol L^−1^ Cyanex 923 and varying HPMBP concentrations. The aqueous phase contained 0.01 mol L^−1^ MgCl_2_ and 0.01 mol L^−1^ LiCl, buffered at pH about 5.35 using 0.20 mol L^−1^ acetate buffer solutions. The O/A ratio was 1/1, at room temperature (22°C) [Color figure can be viewed at wileyonlinelibrary.com]

The synergism between β‐diketones and Cyanex 923 for Li extraction has been reported by many studies, but the synergism for Mg extraction is not known yet. We first investigated the extraction of both Li and Mg by mixtures of HPMBP (as a representative of β‐diketones) and Cyanex 923 dissolved in *p*‐cymene, with results shown in Figure 2. When the concentration of HPMBP was fixed, the extraction of both Mg and Li increased with increasing concentration of Cyanex 923. The enhanced extraction is evidence of synergism for the extraction of both metals. The synergism factor *R*_Mg_ under the condition of 0.10 mol L^−1^ HPMBP and 0.10 mol L^−1^ Cyanex 923 is >1,000. By contrast, *R*_Li_ is only 6.8 under the same condition. The comparison clearly shows that the synergism for Mg extraction is much stronger than that for Li extraction. The percentage extraction of Mg is significantly higher than Li, with a separation factor of *α*_Mg/Li_ > 1,000 at 0.03 mol L^−1^ Cyanex 923. It should be noted that when Cyanex 923 was <0.02 mol L^−1^, the formation of a white precipitate was observed at the interface. According to the mass balance of metals in the two phases, the precipitate is Mg, which is possibly a PMBP‐Mg complex. Therefore, HPMBP alone can react with Mg but the complex is not soluble in either phase. However, Cyanex 923 alone does not extract either Mg or Li. When the concentration of Cyanex 923 was fixed, the extraction of both Mg and Li increased with increasing concentration of HPMBP, also supporting the synergism between HPMBP and Cyanex 923 for the extraction of Mg and Li.

### 3.2 Effect of pH

**FIGURE 3:**
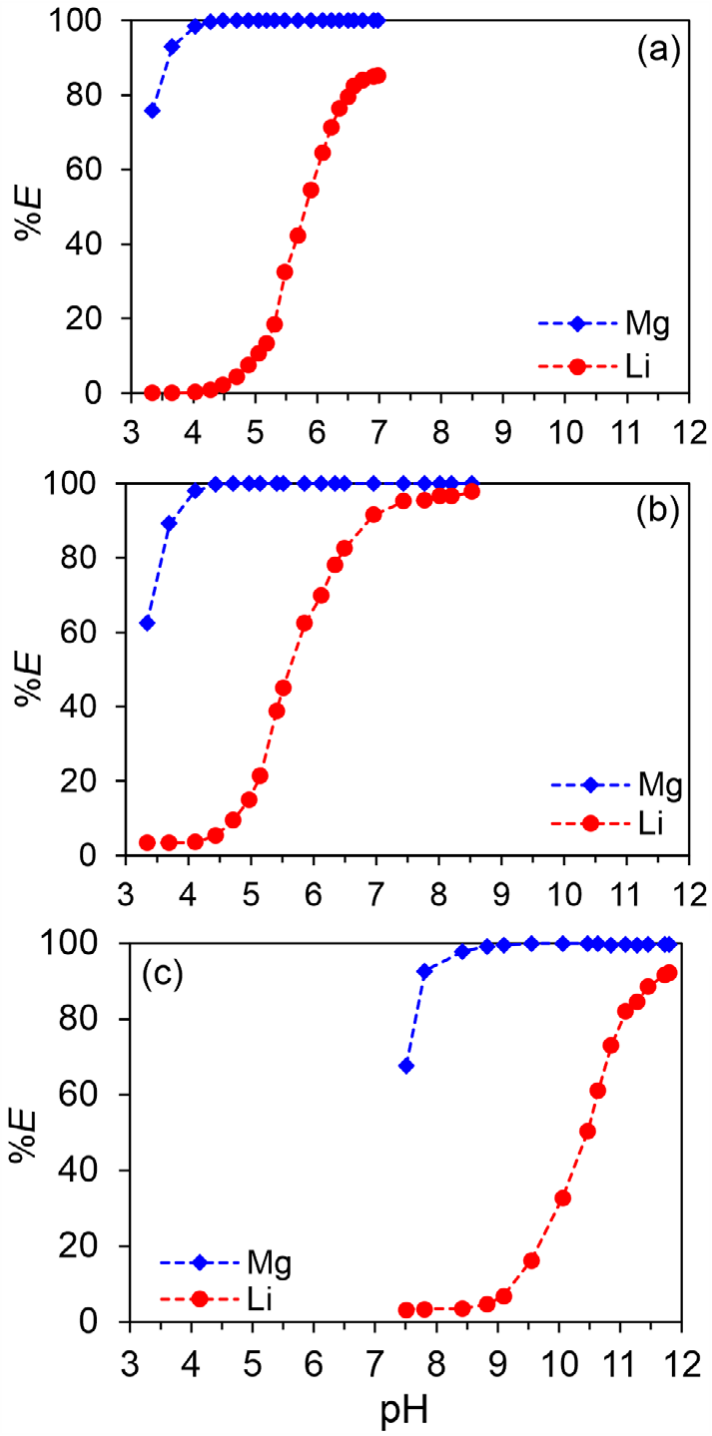
Effect of pH on the extraction of Mg and Li by the synergic SX systems: (a) 0.10 mol L^−1^ HPMBP and 0.10 mol L^−1^ Cyanex 923; (b) 0.10 mol L^−1^ HTTA and 0.10 mol L^−1^ Cyanex 923; (c) 0.10 mol L^−1^ HDBM and 0.10 mol L^−1^ Cyanex 923. Aqueous solutions were 0.01 mol L^−1^ MgCl_2_ and 0.01 mol L^−1^ LiCl buffered by 0.20 mol L^−1^ acetate, MES, Tris, or methylamine buffer solutions in appropriate pH range. The O/A ratio was 1/1, at room temperature (22°C) [Color figure can be viewed at wileyonlinelibrary.com]

The effect of the pH on the extraction of Mg and Li was studied for all the three β‐diketone (HPMBP, HTTA, and HDBM) synergic SX systems (Figure 3). Buffer solutions were used to help control the pH to the desired values. All the pH isotherms are smooth, despite using several buffers, indicating that the effect of the buffer on the extraction is negligible. When the pH was as low as about 3.3, up to 75% Mg was extracted by the HPMBP system. The extraction of Mg was complete when pH > 4.5. The extraction of Li by the HPMBP system was negligible when pH < 4 and increased with increasing pH until reaching a plateau of about 85% at pH > 6.7. Only 2.3% of Li was extracted at pH 4.5, where Mg extraction was quantitative. The separation factor (*α*_Mg/Li_) was >20,000 at pH 4.04 and 4.28, where Mg extraction was not complete yet and Li extraction was detectable. The very large *α*_Mg/Li_ value means excellent separation of the two metals.

In terms of the HTTA synergic SX system, it is interesting to notice that the extraction of Mg was similar to the isotherm of the HPMBP system, although HTTA (pK_a_ = 6.25) has a higher pK_a_ value than HPMBP (pK_a_ = 3.92). The extraction of Li was low when pH < 4.5 and increased rapidly when pH > 4.5. At pH 8.5, up to 98% of Li was extracted. At pH 4.7 where Mg extraction was just complete, the extraction of Li was 9.5%. The *α*_Mg/Li_ at pH 4.11 and 4.45 was about 1,400 and 6,400, respectively.

The effective pH of the HDBM synergic SX system was much higher than the pH of the other two systems. The extraction of Mg was not complete until the pH was about 9.5, where the extraction of Li was about 16%, higher than the other two systems. The highest Li extraction of about 92% was reached at pH 11.8. The *α*_Mg/Li_ at pH 8.43, 8.83, and 9.10 were about 1,200, 2,800, and 2,900, respectively. It should be noted that a small amount of white precipitate was observed when pH > 9.5, which will be discussed in the following sections.

In short, all three β‐diketone synergic SX systems have good Mg/Li selectivity with *α*_Mg/Li_ > 1,000. The HPMBP synergic SX system has the highest *α*_Mg/Li_ and the lowest co‐extraction of Li at the pH value where the Mg extraction was just complete, and hence has the best selectivity. The HTTA synergic SX system is the most efficient for Li extraction. The extraction of Mg using HDBM has the risk of forming a precipitate due to its high pKa.

### 3.3 Comparison with Versatic Acid 10

**FIGURE 4:**
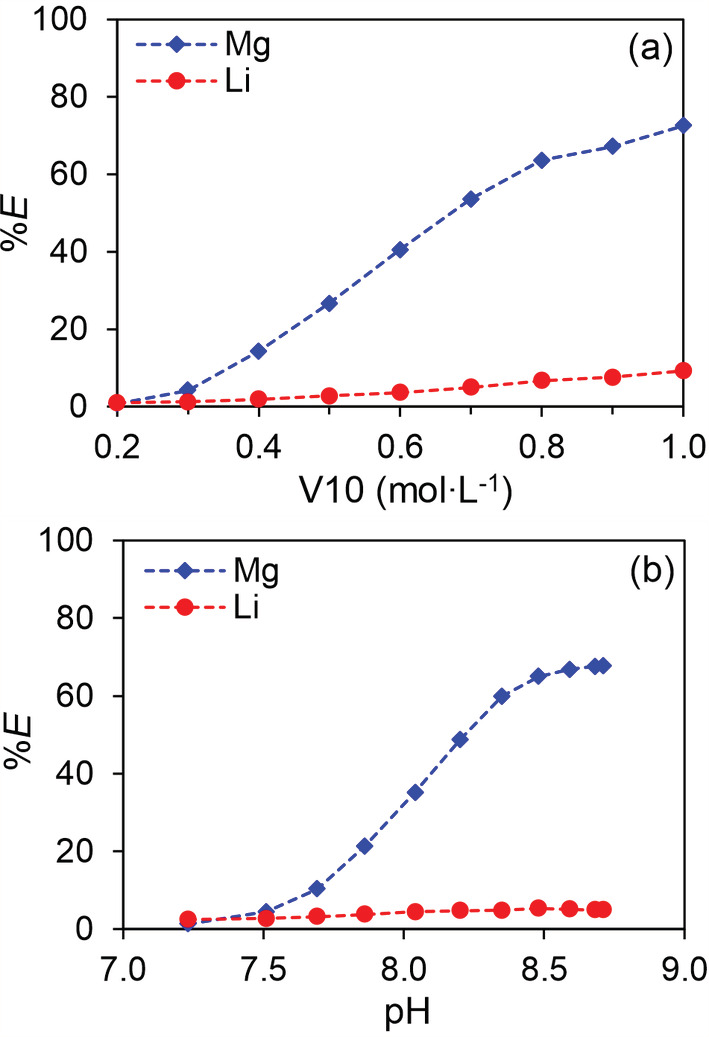
Extraction of Mg and Li by Versatic Acid 10 (V10): (a) the effect of V10 concentration, equilibrium pH was around 8.15; (b) the effect of pH when the V10 concentration was 0.60 mol L^−1^. The aqueous solution contained 0.01 mol L^−1^ MgCl_2_ and 0.01 mol L^−1^ LiCl buffered by 1.0 mol L^−1^ Tris buffer solution. The O/A ratio was 1/1, at room temperature (22°C) [Color figure can be viewed at wileyonlinelibrary.com]

Here we compare the performance of the β‐diketone synergic SX systems for Mg and Li separation with the Versatic Acid 10 system (Figure 4) because the latter has been found to have good Mg/Li selectivity as discussed above. Below 0.20 mol L^−1^ of Versatic Acid 10, the extraction of both Mg and Li was <1%. With the increase of Versatic Acid 10 concentration in *p*‐cymene, the extraction of Mg and Li increased and reached the highest values at 1.0 mol L^−1^ Versatic Acid 10, where 73% Mg and 9.2% Li was extracted. The highest *α*_Mg/Li_ was only 26, observed at 1.0 mol L^−1^ Versatic Acid 10.

Higher Versatic Acid 10 concentration did not improve the separation much, hence 0.60 mol L^−1^ Versatic Acid 10 was selected to study the pH effect. At pH 7.5, 4.4% Mg and 2.7% Li were extracted, respectively. With increasing pH, the extraction of Mg increased until reaching a plateau of about 67% at pH > 8.5, while the extraction of Li only slightly increased to about 5%. The highest *α*_Mg/Li_ was about 40, obtained at pH 8.7. Compared with the β‐diketone synergic SX systems, where 100% Mg could be extracted with *α*_Mg/Li_ > 1,000, the Versatic Acid 10 system considerably underperformed in terms of both Mg extraction efficiency and Mg/Li selectivity.

### 3.4 Removal of Mg for Li purification

The pH isotherms have shown that β‐diketone synergic SX systems have high Mg extraction efficiency and high Mg/Li selectivity. Here we studied the removal of Mg impurity from concentrated LiCl solutions. A synthetic solution containing 24 g L^−1^ Li and 0.24 g L^−1^ Mg (about 1 wt% Mg impurity) was used as a feed solution. This composition is comparable to the solution in the study of Virolainen et al.: 26.0–34.0 g L^−1^ Li, 1.14–1.55 g L^−1^ Ca and 0.045–0.075 g L^−1^ Mg.^16^ The feed LiCl solution in the current study did not contain Ca because the removal of Ca is easier than Mg since Ca is more affinitive to extractants.^16^, ^18^ The effect of Ca on the removal of Mg will be considered in further studies where Ca is included in the feed solution.

Acidic extractants, including β‐diketones and Versatic Acid 10, require adjustment of pH during extraction because protons are released which would hinder the extraction. In this study, we simply added certain amounts of LiOH to neutralize the released proton instead of controlling the pH. It should be noted that after the addition of LiOH to the aqueous solution, a white Mg(OH)_2_ precipitate was observed. The precipitate completely disappeared after extraction for the Versatic Acid 10 system, was negligible for the HPMBP and the HTTA system but was clearly observable for the HDBM system. The precipitate in the HDBM system might be a mixture of Mg(OH)_2_ (due to the low acidity of HDBM) and a DBM‐Li complex. The good mass balance of Mg (94–105%) in the two phases of the HDBM system indicates that the precipitate was mainly a DBM‐Li complex.

**FIGURE 5:**
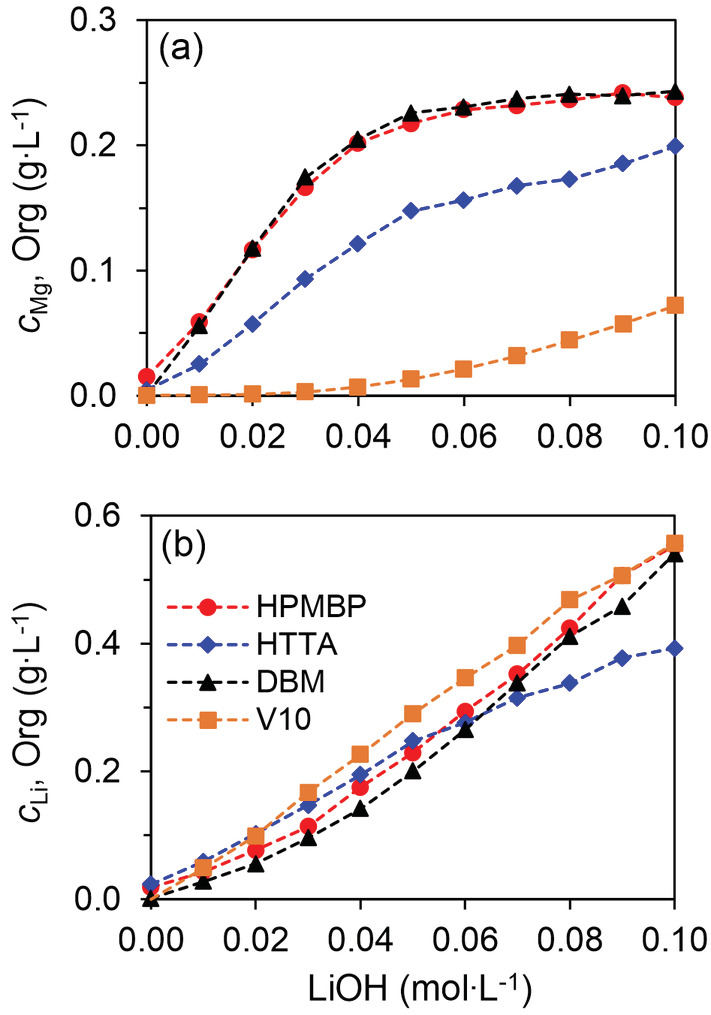
Selective removal of Mg from Li‐rich aqueous solutions by various extraction systems. (a) Loading of Mg into the organic phase; (b) co‐extraction of Li into the organic phase. The aqueous solution was 0.24 g L^−1^ Mg and 24 g L^−1^ Li with varying LiOH concentration; the organic solutions were: 0.10 mol L^−1^ β‐diketone (HPMBP, HTTA or HDBM) and 0.10 mol L^−1^ Cyanex 923, or 0.60 mol L^−1^ V10. The O/A ratio was 1/1, at room temperature (22°C) [Color figure can be viewed at wileyonlinelibrary.com]

The extraction of Mg and Li with various amounts of LiOH for the three β‐diketone synergic SX systems and the Versatic Acid 10 system is presented in Figure 5. The extraction of both Mg and Li was enhanced with larger amounts of LiOH for all of the four SX systems, but the four systems also differ significantly in terms of extraction efficiency and selectivity. The HPMBP and the HDBM system had the highest Mg extraction efficiency, extracting 228.3 mg L^−1^ (91.3%) and 230.5 mg L^−1^ Mg (92.6%) at 0.06 mol L^−1^ LiOH, and further reaching about 238.1 mg L^−1^ (97.3%) and 243.2 mg L^−1^ Mg (about 97.7%) at 0.10 mol L^−1^ LiOH, leaving only 6.5 mg L^−1^ and 5.7 mg L^−1^ Mg in the raffinate. However, the HTTA system extracted only 199.0 mg L^−1^ Mg (78.7%) at 0.10 mol L^−1^ LiOH, leaving 54.0 mg L^−1^ Mg in the raffinate. The lower removal of Mg by the HTTA system might be due to the higher solubility of HTTA in the aqueous solution (Figure S2). By contrast, Versatic Acid 10 only extracted 71.9 mg L^−1^ Mg (about 28.9%) at 0.10 mol L^−1^ LiOH, leaving176.8 mg L^−1^ Mg in the raffinate, significantly underperforming the β‐diketone synergic SX systems.

On top of a high Mg extraction, we also want to have a low co‐extraction of Li. The co‐extraction of Li by the HPMBP, HDBM and the Versatic Acid 10 systems were comparable: about 550 mg L^−1^ Li was co‐extracted at 0.10 M LiOH (about 2.2%). The HTTA system had a lower co‐extraction of Li (about 390 mg L^−1^ and corresponds to about 1.5%). The lower Li extraction can also be explained by the loss of HTTA to the aqueous solution due to its higher solubility. A comparison of the four SX systems clearly shows that the HPMBP and the HDBM synergic SX systems have the best Mg extraction efficiency and good Mg/Li selectivity for Li purification. However, HDBM has the risk of forming a precipitate. As a result, the HPMBP system is chosen for further optimization of the separation process.

### 3.5 β‐Diketone precipitates

**FIGURE 6:**
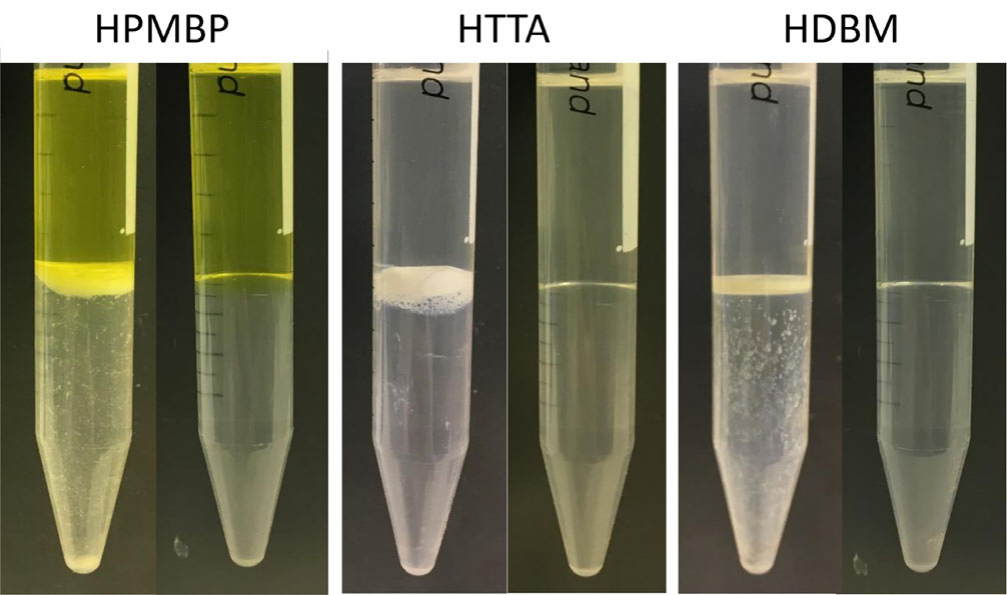
Extraction of Mg from 0.05 mol L^−1^ MgCl_2_ and 0.01 mol L^−1^ LiOH by 0.10 mol L^−1^ HPMBP, HTTA or HDBM without (left) and with (right) 0.10 mol L^−1^ Cyanex 923 [Color figure can be viewed at wileyonlinelibrary.com]

In addition to the Mg(OH)_2_ and HDBM‐Li precipitates discussed above, it is worth discussing the precipitation of β‐diketone‐Mg. The extraction of Li and Mg at about pH 5.3 by HPMBP generated a white precipitate when the Cyanex 923 concentration was low (Figure 2a), and the mass balance showed a loss of 99.3% and 26.4% of Mg at 0.00 and 0.01 mol L^−1^ Cyanex 923, but no loss of Li. Therefore the white precipitate is a PMBP‐Mg complex. To test whether all β‐diketones form precipitates with Mg, 0.10 mol L^−1^ HPMBP, HTTA and HDBM, respectively, were contacted with 0.05 mol L^−1^ MgCl_2_ solution with the addition of 0.01 mol L^−1^ LiOH to neutralize protons. A white precipitate was observed at the interface for all the three β‐diketones (Figure 6). It should be noted that the addition of 0.01 mol L^−1^ LiOH alone did not generate a precipitate. This suggests that the white precipitates are β‐diketone‐Mg complexes and they are poorly soluble in either the organic phase or the aqueous phase. The disappearance of the white precipitate after the addition of 0.10 mol L^−1^ Cyanex 923 is most likely due to the formation of the β‐diketone‐C923‐Mg complexes that are much more soluble in the organic phase than the β‐diketone‐Mg complexes.

We have observed β‐diketone‐Li precipitation and β‐diketone‐Mg precipitation, both of which can be avoided by the addition of Cyanex 923 to form more soluble complexes. In addition, Mg(OH)_2_ precipitation may occur if the pH is too high. To avoid Mg(OH)_2_ precipitation, β‐diketone (acidic agents) concentration should be higher than LiOH. These considerations are taken into account in the following process development.

### 3.6 Extraction mechanism

#### 3.6.1 Slope analysis

**FIGURE 7:**
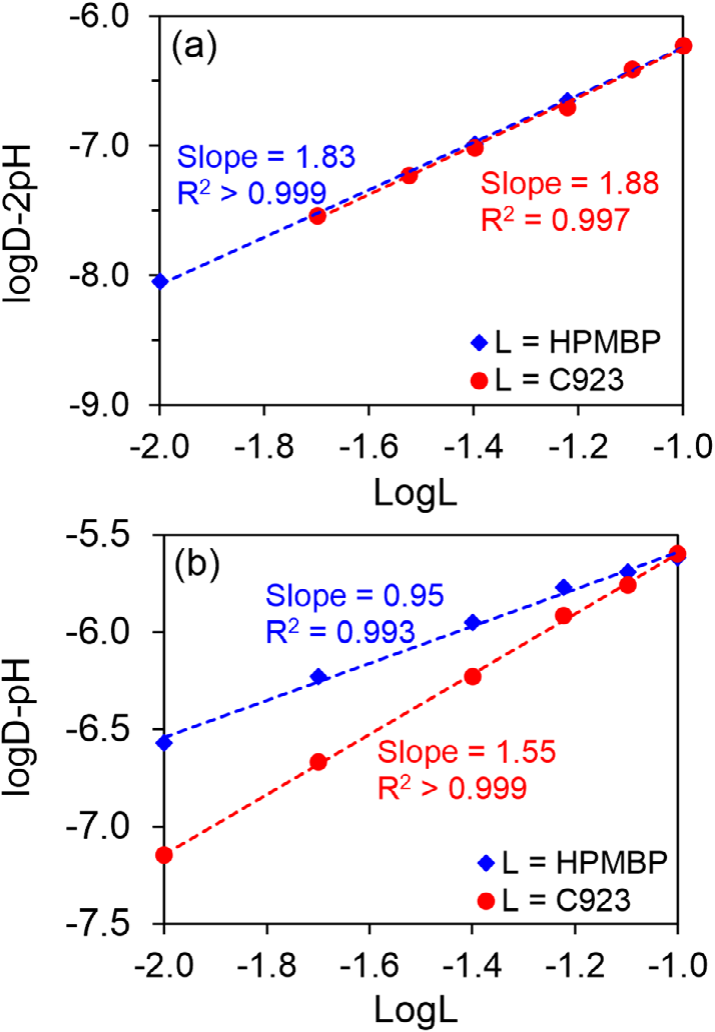
Slope analysis for the extraction of Mg (a) and Li (b) by mixtures of HPMBP and Cyanex 923. The aqueous solutions contained 0.01 mol L^−1^ MgCl_2_ buffered at pH about 3.5 using 0.20 mol L^−1^ formate buffer solution (for Mg) or 0.01 mol L^−1^ LiCl buffered at pH about 6.1 using 0.20 mol L^−1^ MES buffer solution (for Li). The concentration of HPMBP (or Cyanex 923) in the organic phase was 0.10 mol L^−1^ while changing the concentration of Cyanex 923 (or HPMBP) [Color figure can be viewed at wileyonlinelibrary.com]

Before optimizing the process for Mg removal, it is important to understand how Mg and Li are extracted by the β‐diketone synergic SX systems. Slope analysis for Mg extraction showed slopes of close to 2 for both HPMBP and Cyanex 923 (Figure 7a), indicating the formation of a complex of the stoichiometry of [Mg∙A_2_∙(C923)_2_] (A^−^ donates deprotonated HPMBP). The formation of this complex is quite reasonable, because two HPMBP molecules are needed to neutralize the charge of Mg^2+^, and Mg^2+^ often exhibits a coordination number of six (HPMBP is bidentate and Cyanex 923 is monodentate). Slope analysis for Li extraction gave a slope of close to 1 for HPMBP, and a slope of 1.55 for Cyanex 923 (Figure 7b). Solely based on the slope analysis, the complex can be supposed to be [Li∙A∙(C923)_1.55_]_._ There are several slope analysis studies for Li extraction by β‐diketones and TOPO, in which slopes between 1.3 and 2 have been reported. These slopes are often explained as forming a mixture of complexes coordinating with one and two TOPO molecules.^35^

#### 3.6.2 Maximum loading

**FIGURE 8:**
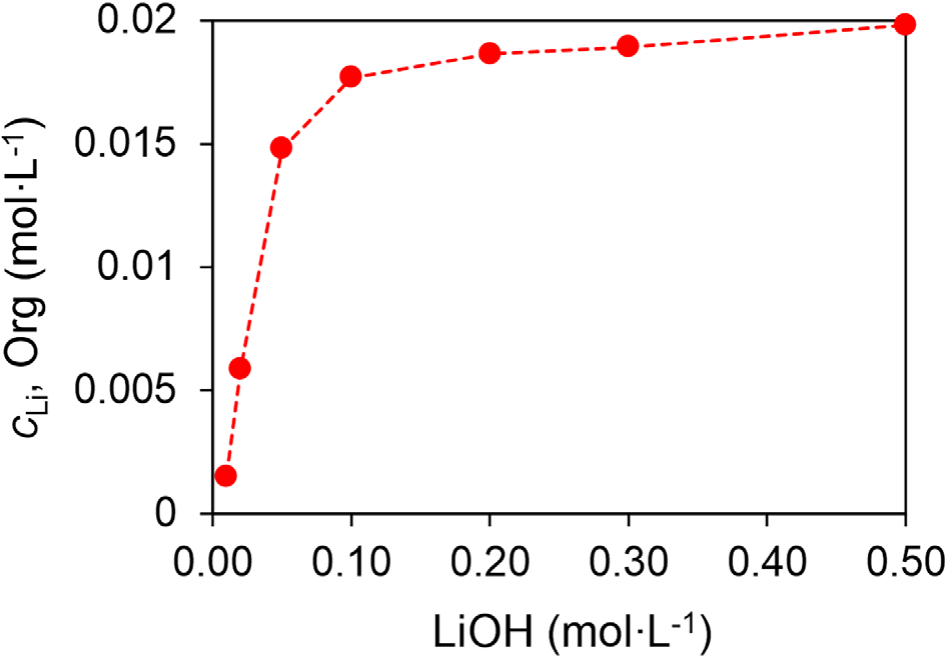
Loading of Li to 0.10 mol L^−1^ HPMBP and 0.02 mol L^−1^ Cyanex 923 with various initial LiOH concentrations (horizontal axis). Data for LiOH >0.50 mol L^−1^ are not shown because precipitation occurred. The O/A ratio was 1/1, at room temperature (22°C) [Color figure can be viewed at wileyonlinelibrary.com]

To further investigate the speciation of Li extraction, loading of Li to the organic phase was conducted with excess HPMBP (0.10 mol L^−1^) in the organic phase and 0.02 mol L^−1^ Cyanex 923, while the aqueous phase had excess LiOH (Figure 8). The result showed that >0.018 mol L^−1^ Li was loaded to the organic phase, yielding a C923:Li ratio of about 1:1. A similar loading result was obtained by buffering the aqueous solution (Figure S3). Besides, at the maximum loading of Li in Figure 5b, the ratio of C923:Li was about 1.03:1, very close to 1:1. Under this condition, almost all the HPMBP (or HDBM) and Cyanex 923 had been consumed for Mg and Li extraction. These results mean that a complex of the stoichiometry of [Li∙A∙(C923)] can be formed, although the formation of [Li∙A∙(C923)_2_] cannot be ruled out when Cyanex 923 is present in excess.

#### 3.6.3 ^7^Li NMR spectra

**FIGURE 9:**
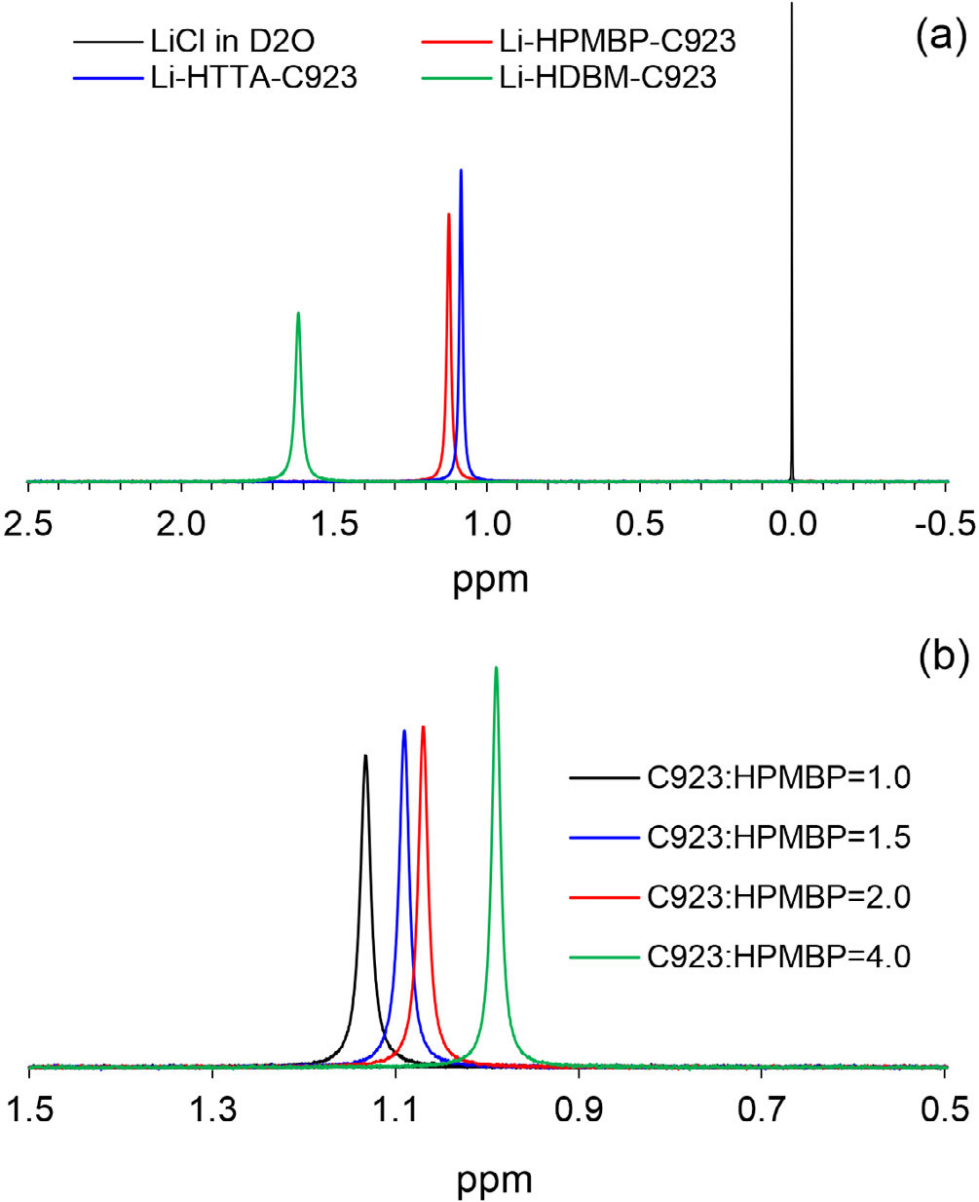
^7^Li NMR spectra of the organic phase: (a) 0.10 mol L^−1^ β‐diketone and 0.20 mol L^−1^ Cyanex 923 loaded with Li (about 0.085 mol L^−1^); (b) Organic phase with varying C923:HPMBP ratios (0.05 mol L^−1^ HPMBP) loaded with Li (about 0.043 mol L^−1^). Note that the organic phase was not mixed with a deuterated solvent, but a capillary tube containing D_2_O was inserted into the NMR tube for locking the frequency [Color figure can be viewed at wileyonlinelibrary.com]

^7^Li NMR spectrum is a useful probe to study the speciation of Li in solvent extraction.^40^ ^7^Li NMR spectra were recorded for the organic phase loaded with Li (Figure 9a). The spectrum of 0.10 mol L^−1^ LiCl in D_2_O was set as a reference for shift (0 ppm). The peak in this reference spectrum is very narrow and sharp, but the peaks in the spectra of the organic solutions are much broader. The broad spectra are an indication of containing either low‐symmetry species or a mixture of similar species. This observation is consistent with the above discussions that Li is extracted as a mixture of two species. It is also interesting to note that the spectra of Li‐PMBP‐C923 and Li‐TTA‐C923 have similar shifts, while the spectrum of Li‐DBM‐C923 is more down‐field shifted. This difference in shift might be explained by the different pK_a_ values of β‐diketones. As shown in pH isotherms (Figure 3), HPMBP and HTTA extract Li in similar pH ranges (5–7), while HDBM requires a much higher pH (>9).

To further investigate the speciation of Li in the loaded organic phase, Li was loaded to the organic phase with varying C923‐to‐HPMBP ratios and the corresponding ^7^Li NMR spectra were recorded (Figure 9b). It was found that the spectra shifted from 1.13 to 0.99 ppm as the C923‐to‐HPMBP ratio changed from 1.0 to 4.0. The movement of the spectra indicates the change of Li speciation. When the C923‐to‐HPMBP ratio was 1.0, the dominating species might be [Li∙PMBP∙(C923)]; as the ratio increased, more [Li∙HPMBP∙(C923)_2_] is probably formed. In summary, a mixture of [Li∙HPMBP∙(C923)_*x*_] (*x* = 1, 2) is formed in the loaded organic phase and the dominating species depends on the C923‐to‐HPMBP ratio.

### 3.7 Optimization of the separation

#### 3.7.1 Effect of Cyanex 923 concentration

**FIGURE 10:**
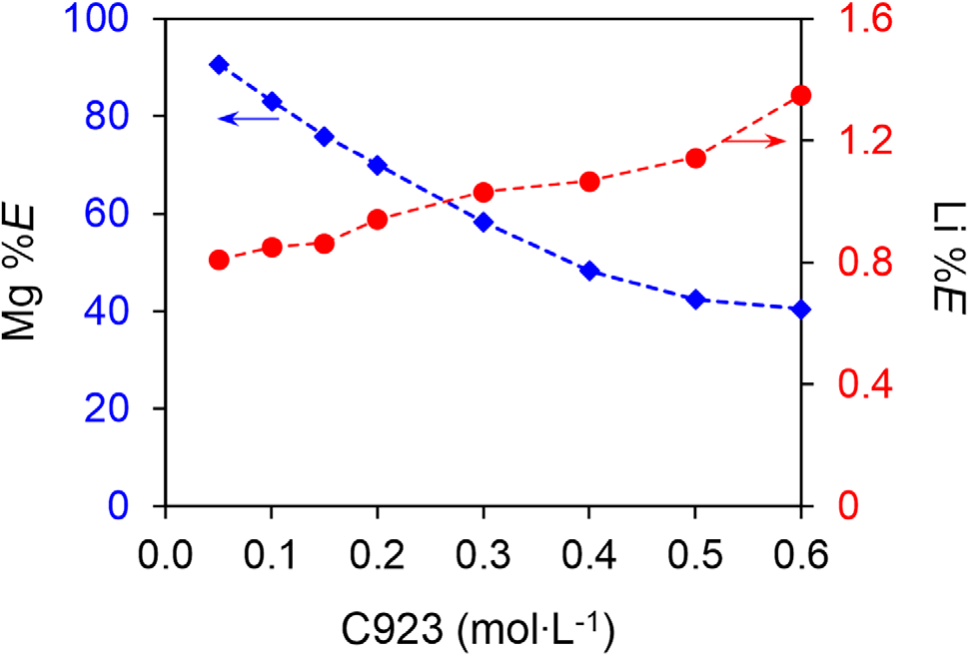
Effect of Cyanex 923 on the extraction of Mg and Li. The organic phase: 0.06 mol L^−1^ HPMBP and various Cyanex 923 concentration; the aqueous phase: 24 g L^−1^ Li, 0.24 g L^−1^ Mg and 0.05 mol L^−1^ LiOH. The O/A ratio was 1/1, at room temperature (22°C) [Color figure can be viewed at wileyonlinelibrary.com]

Mg and Li are extracted by HPMBP and Cyanex 923 in different stoichiometric ratios, therefore the HPMBP:C923 ratio might affect the selectivity. The effect of the Cyanex 923 concentration on the extraction of Mg and Li has been studied and presented in Figure 10. The amount of HPMBP and LiOH were reduced from 0.10 to 0.06 mol L^−1^ and 0.05 mol L^−1^, respectively, to suppress the co‐extraction of Li. Interestingly, the extraction of Li increased from 200 mg L^−1^ (0.8%) at 0.05 mol L^−1^ Cyanex 923 to 305 mg L^−1^ (1.3%) at 0.60 mol L^−1^ Cyanex 923; but the extraction of Mg reduced from 225 mg L^−1^ (91%) at 0.05 mol L^−1^ Cyanex 923 to 96 mg L^−1^ (41%) at 0.60 mol L^−1^. The opposite trend for Li and Mg extraction is due to the competition for HPMBP, which was the limiting reagents in the organic phase. On the basis of the amount of Li and Mg extracted, the amount of HPMBP consumed fluctuated in the range of 0.046–0.052 mol L^−1^, which is close to the amount of LiOH added (0.05 mol L^−1^). This calculation means that virtually all of the deprotonated HPMHP was consumed for the extraction of Mg and Li, hence Mg and Li competed for HPMBP. Importantly, the enhanced extraction of Li at higher Cyanex 923 concentration might be explained by the higher C923:HPMBP ratio exhibited for Li extraction (1.55 based on slope analysis) than that for Mg extraction (about 1.0 based on slope analysis). Therefore, higher Cyanex 923 concentration is preferential for Li extraction. To reduce the co‐extraction of Li while maintaining efficient Mg extraction, a C923:HPMBP ratio of 1:1 is recommended.

#### 3.7.2 Effect of Mg concentration

**FIGURE 11:**
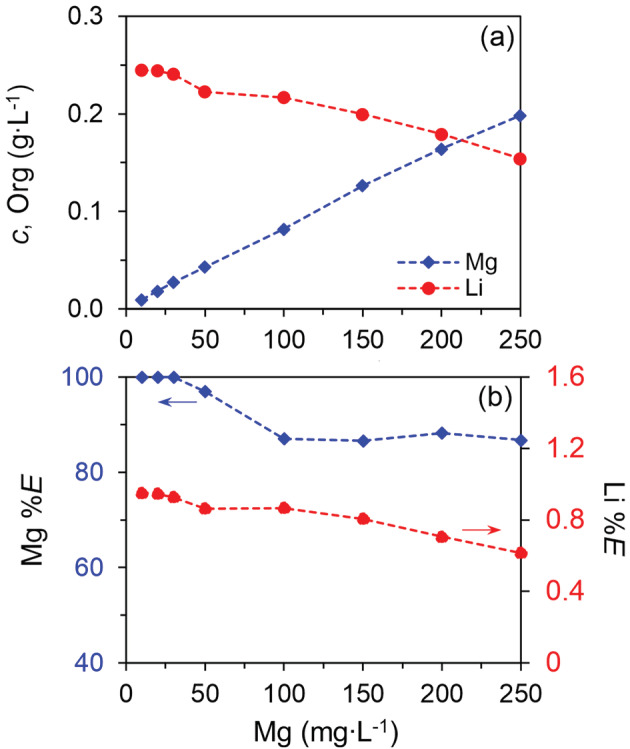
Effect of initial Mg concentration on the extraction of Mg and Li. Organic phase: 0.06 mol L^−1^ HPMBP and 0.06 mol L^−1^ Cyanex 923; aqueous phase: 24 g L^−1^ Li, 0.04 mol L^−1^ LiOH and various Mg concentrations. The O/A ratio was 1/1, at room temperature (22°C) [Color figure can be viewed at wileyonlinelibrary.com]

The extraction of Mg must be studied under different Mg concentrations, because Mg could not be removed completely in one extraction step and the remaining Mg must be extracted by additional extraction steps. In this section, we further reduced the LiOH concentration from 0.05 to 0.04 mol L^−1^ to suppress the co‐extraction of Li, because we found that all the deprotonated HPMBP was consumed for Li and Mg extraction. The results in Figure 11 show that the higher Mg concentration in the aqueous phase, the higher loading in the organic phase. Moreover, the higher Mg loading scrubs the extraction of Li and the extraction of Li was <1.0% in all cases. When the Mg concentration in the aqueous feed was between 100 and 250 mg L^−1^, about 87% Mg could be extracted; when the Mg was <50 mg L^−1^, > 95% Mg can be extracted. Therefore, a second extraction stage would remove the majority of Mg that was left in the aqueous solution after the first extraction stage (<50 mg L^−1^), resulting in <2 mg L^−1^ Mg in the raffinate. If a third extraction stage would be used, the Mg should be removed completely (<0.1 mg L^−1^) and the co‐extraction of Li can be expected to be <1.0% since Mg scrubs Li.

### 3.8 Batch counter‐current extraction

#### 3.8.1 McCabe‐Thiele diagram

**FIGURE 12:**
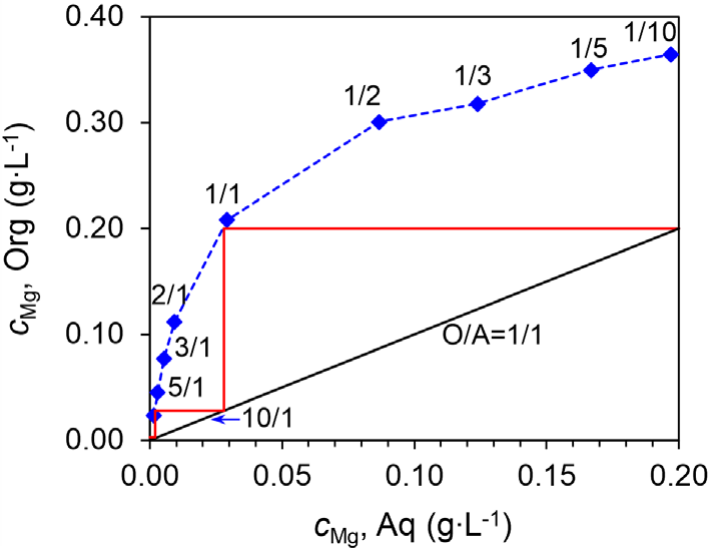
McCabe‐Thiele diagram for Mg extraction under various O/A ratios at room temperature (22°C). Metal concentrations in the aqueous and the organic phase were determined at extraction equilibria. Organic phase: 0.06 mol L^−1^ HPMBP and 0.06 mol L^−1^ Cyanex 923; aqueous phase: 0.24 g L^−1^ Mg, 24 g L^−1^ Li, and 0.04 mol L^−1^ LiOH [Color figure can be viewed at wileyonlinelibrary.com]

To estimate the number of extraction stages needed for the complete removal of Mg, the effect of phase ratio was studied and the McCabe‐Thiele diagram was constructed (Figure 12). Based on the diagram, a phase ratio of 1/1 was chosen for operation, because a higher phase ratio would co‐extract more Li while a lower phase ratio would need too many extraction stages. It was found that two extraction stages would be able to remove the majority of Mg, leaving <3 mg L^−1^ Mg in the raffinate, and a third extraction stage would be able to remove Mg completely.

#### 3.8.2 Batch counter‐current extraction experiment

**FIGURE 13:**
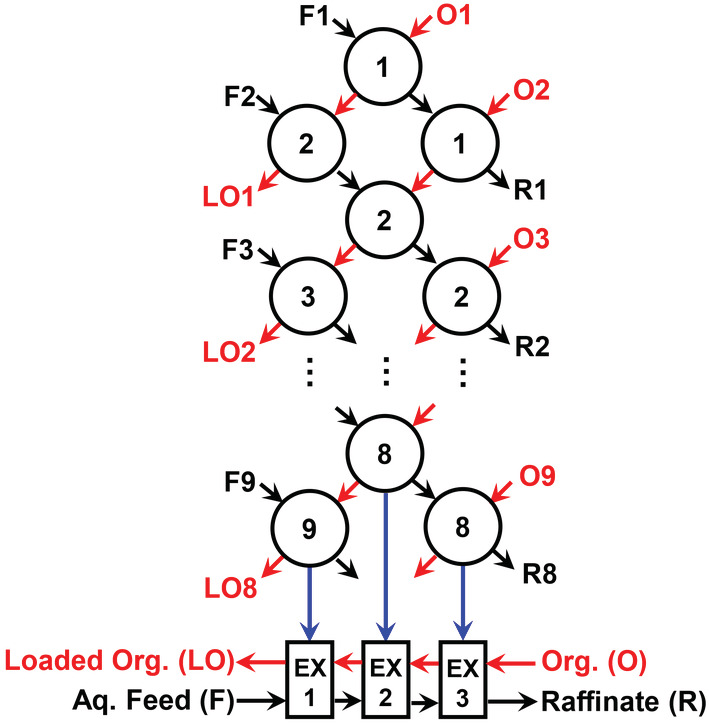
Flow sheet of the three‐stage batch counter‐current extraction experiment. Feed solution (F), organic phase (O), loaded organic phase (LO), raffinate (R) and three extraction stages (EX1, EX2, and EX3) are labeled. Note that F1, F2, …, F9 are the same feed solution [Color figure can be viewed at wileyonlinelibrary.com]

A three‐stage batch counter‐current extraction was conducted to simulate a continuous extraction experiment using a phase ratio of 1/1 according to the McCabe‐Thiele diagram. The flow sheet is shown in Figure 13. LiOH was used to neutralize protons of HPMBP in the above discussions, but Mg(OH)_2_ precipitate induced by LiOH may hinder the continuous extraction operation. Instead of adding LiOH to the aqueous feed solution, the organic phase was first loaded with Li from an aqueous solution containing 24 g L^−1^ Li and 0.04 mol L^−1^ LiOH (without Mg). Li in the loaded organic phase was determined to be 0.27 g L^−1^ (0.039 mol L^−1^), indicating that almost all the deprotonated HPMBP (0.04 mol L^−1^) was consumed for Li extraction. The loaded organic phase was then used for Mg extraction. In principle, Mg would be extracted by replacing (scrubbing) the loaded Li since Mg is more affinitive to the extractants. This extraction is the same as the addition of LiOH to the aqueous feed solution, but avoids the formation of Mg(OH)_2_ precipitate.

**FIGURE 14:**
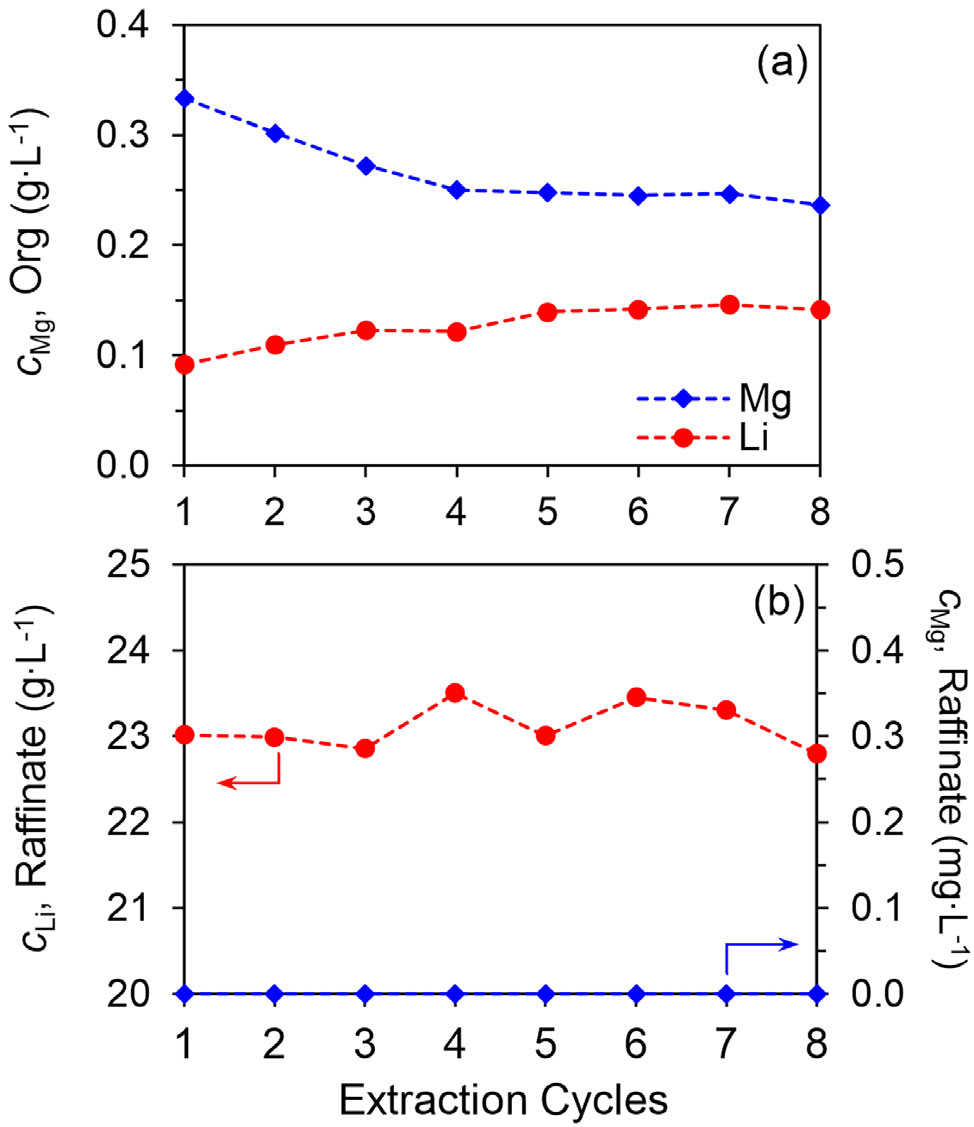
Metals in the loaded organic phase (a) and the raffinate (b) in different extraction cycles of the batch counter‐current extraction experiment. Organic phase: 0.06 mol L^−1^ HPMBP and 0.06 mol L^−1^ Cyanex 923 loaded with 0.27 g L^−1^ Li; aqueous phase: 24 g L^−1^ Li, 0.24 g L^−1^ Mg and without LiOH [Color figure can be viewed at wileyonlinelibrary.com]

Up to 8 extraction cycles were conducted to ensure that a steady state was obtained. The concentrations of Mg and Li in the loaded organic phase and the raffinate in the entire 8 cycles have been presented in Figure 14. In the fifth extraction cycle, the loading of Mg and Li was 248 and 140 mg L^−1^ (about 0.6%), respectively, and the loading was stable in the following extraction cycles. Concerning the raffinates, Li slightly fluctuated around 23 g L^−1^ and Mg was not detectable (<0.1 mg L^−1^) in any extraction cycle. According to the concentrations of metals in the loaded phases and the raffinates, we can safely conclude that after 5 extraction cycles the steady state was obtained and Mg was completely removed (100%) with co‐extraction of about 0.6% Li. Suppose the residual Mg is 0.1 mg L^−1^, the resulting Li purity is >99.999%, by far exceeding the requirement of high‐purity Li_2_CO_3_ (99.99%).

**FIGURE 15:**
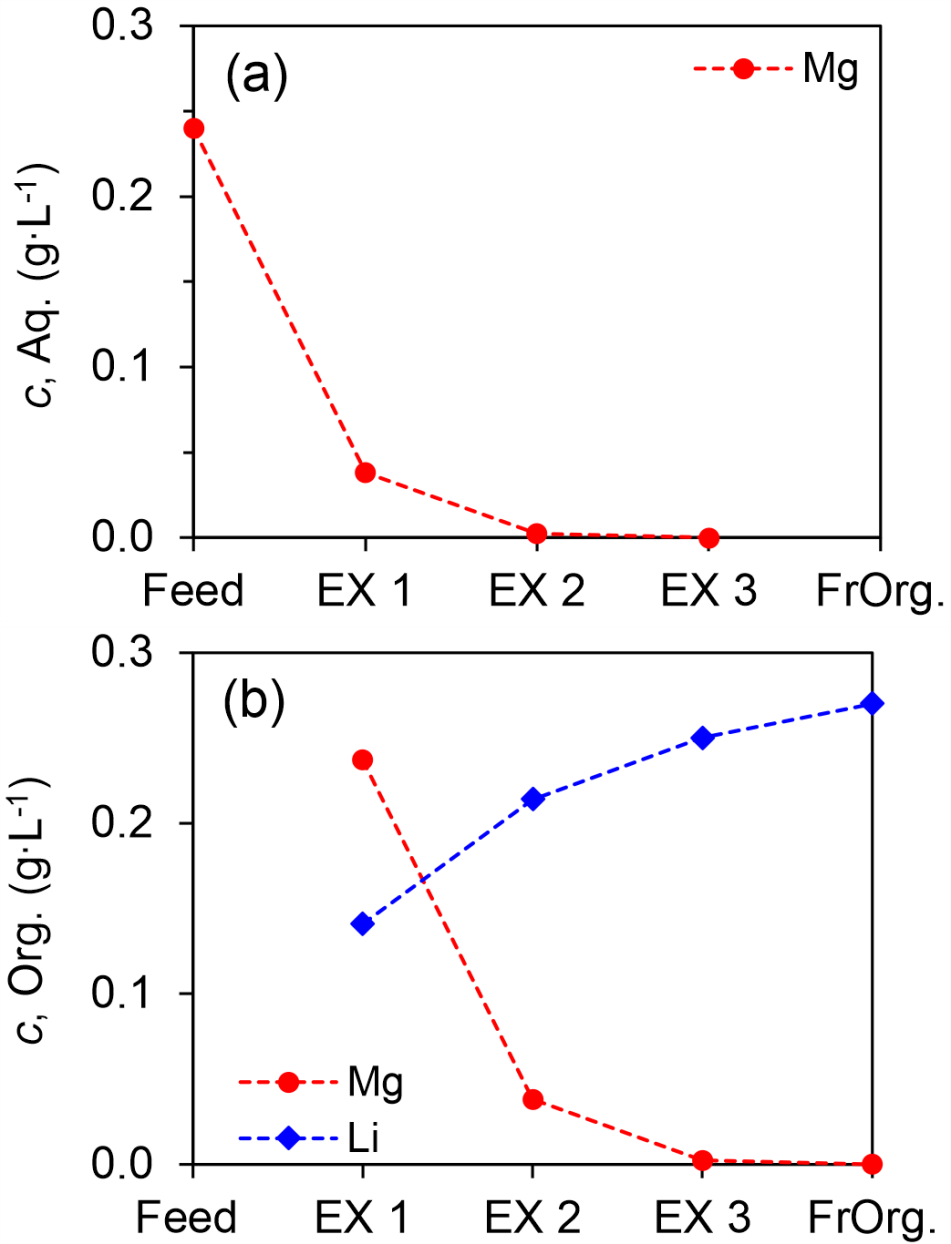
Loading profiles of metals in the three‐stage batch counter‐current extraction of the last extraction cycle. Fresh Organic (FrOrg) solution was preloaded with Li [Color figure can be viewed at wileyonlinelibrary.com]

To understand the batch counter‐current extraction better, we analyzed the loading profiles of metals in the three extraction stages of the eighth extraction cycle (as tagged by arrows in Figure 13). The Li concentration in the aqueous phase was always around 23 g L^−1^ and is not shown in the figure. The Mg concentration was about 240 mg L^−1^ in the feed (Figure 15a), which was reduced to 38 mg L^−1^ after the first extraction stage (EX1) and was further reduced to about 2 mg L^−1^ (< 1%) in the second extraction stage (EX2). Hence, > 99% Mg can be removed in two extraction stages, and the resulting purity of Li is already about 99.99%. In the third extraction stage (EX3), Mg was not detectable and the removal was virtually 100%. The organic phase was originally loaded with 270 mg L^−1^ Li and no Mg (Figure 15b). In the third extraction stage (EX3) about 2 mg L^−1^ Mg was loaded, which is the total amount of Mg that was left in the second extraction stage (EX2); in the second extraction stage (EX2), about 38 mg L^−1^ Mg was loaded; in the first extraction stage (EX1), about 237 mg L^−1^ Mg was loaded, which is more or less the same amount of Mg in the feed solution. As the amount of Mg in the organic phase increased from EX3 to EX1, the amount of Li gradually decreased due to the scrubbing effect of Mg until about 141 mg L^−1^ (0.6%) in EX1. Following the removal of Mg, the loaded organic phase containing about 240 mg L^−1^ and 140 mg L^−1^ Li can be stripped by HCl solution and further separated to recover the co‐extracted Li, potentially using the same HPMBP synergic SX system, but under a different optimized condition.

## 4 CONCLUSIONS

The synergic SX systems containing a β‐diketone (HPMBP, HTTA or HDBM) and Cyanex 923 have remarkable Mg selectivity over Li and considerably outperform the Versatic Acid 10 system (a benchmark) for Mg removal for Li purification. The HPMBP and Cyanex 923 system was selected for the study of the extraction mechanism and process development, because it has a high Mg extraction efficiency and precipitate formation is avoided. Slope analysis, maximum loading and ^7^Li NMR spectra indicate that Mg is extracted as a complex with a stoichiometry of [Mg∙A_2_∙(C923)_2_] (A represents deprotonated β‐diketone) and Li is extracted as a mixture of complexes with stoichiometries of [Li∙A_*x*_∙(C923)_2_] (*x* = 1, 2). A synthetic solution containing 24 g L^−1^ Li and 0.24 g L^−1^ Mg that mimics a concentrated brine solution with Mg impurities was investigated for Mg removal using the HPMBP and Cyanex 923 synergic SX system. In a three‐stage batch counter‐current extraction, Mg was removed virtually completely (100%), with as little as 0.6% co‐extraction of Li. The excellent separation result is the best performance reported so far and the system might be useful in the industry for the production of battery‐grade and high‐purity Li_2_CO_3_.

## CONFLICT OF INTEREST

The authors declare no conflict of interest.

## Supporting information


**Data S1** Supporting Information.
