# Supplementary material for: Selective removal of magnesium from lithium‐rich brine for lithium purification by synergic solvent extraction using β‐diketones and Cyanex 923
Source: AIChE J. 2020 Apr 21;66(7):e16246. doi: 10.1002/aic.16246 (PMC9286357; doi:10.1002/aic.16246)
Supplement: Supplementary file 1 — Data S1 Supporting Information. [file AIC-66-0-s001.pdf]

Supporting Information

Selective removal of magnesium from lithium-rich brine for lithium purification by synergic solvent extraction using β-diketones and Cyanex 923

Zheng Li* and Koen Binnemans

Loss of β-diketones to the aqueous solution

The loss of β-diketones from the organic phase to the aqueous solution was studied by contacting the organic phase (without Cyanex 923) with water and LiOH solutions (Fig. S2). The concentration of β-diketones in the organic phase was determined by ^{1}H NMR. When contacted with aqueous solutions, the losses of HPMBP and HDBM to the aqueous phase were negligible, but about 9% of HTTA was lost to the aqueous phase. With the addition of LiOH, the concentration of all three β-diketones in the organic phase were considerably reduced, with the loss of HTTA being the highest, followed by HPMBP, and HDBM had the smallest loss. HTTA did not show any precipitate and HTTA-Li is the most soluble in water. HPMBP showed a small amount of white precipitate formation at 0.10 mol·L^{-1} LiOH. Concerning HDBM, the addition of 0.02 LiOH generated a clearly visible white precipitate, which is due to the limited solubility of DBM-Li. These observations show that HTTA is the most soluble in water, while DBM is the least soluble. The relatively smaller extraction of Mg and Li by HTTA in Figure 5 might be explained by the higher solubility of HTTA in the aqueous solution. It should be noted that with the addition of Cyanex 923, a more hydrophobic complex of β-diketone-C923-Li would be formed hence significantly reducing the loss of β-diketones to the aqueous phase.

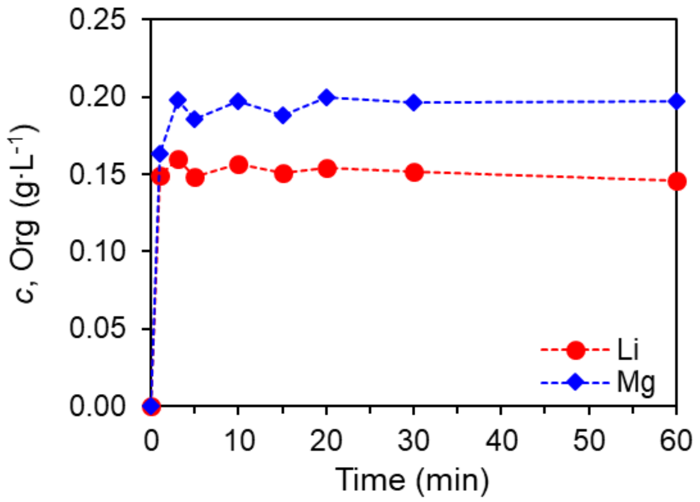

Figure S1. Rate of the extraction of Mg and Li. The organic phase: 0.06 mol·L^{-1} HPMBP and 0.06 mol·L^{-1} Cyanex 923; the aqueous phase: 0.24 g·L^{-1} Mg, 24 g·L^{-1} Li and 0.04 mol·L^{-1} LiOH. The O/A ratio was 1/1, at room temperature (22 °C).

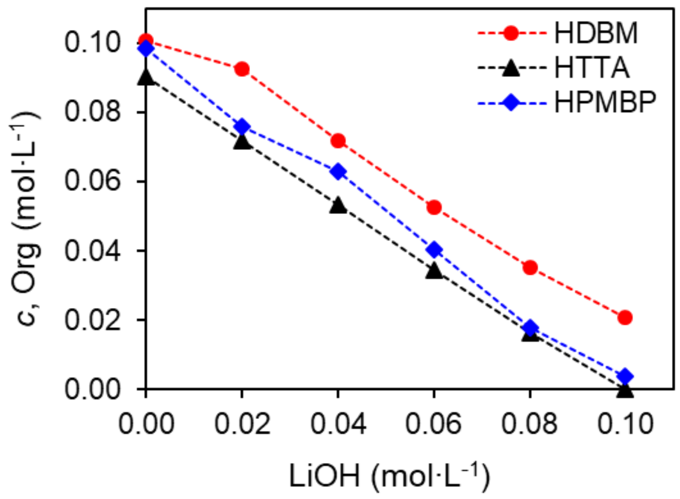

Figure S2. Loss of β-diketones to the aqueous solutions containing LiOH. The organic phase was 0.10 mol·L^{-1} β-diketone (HPMBP, HTTA or HDBM) dissolved in *p*-cymene and the aqueous phase was LiOH solution.

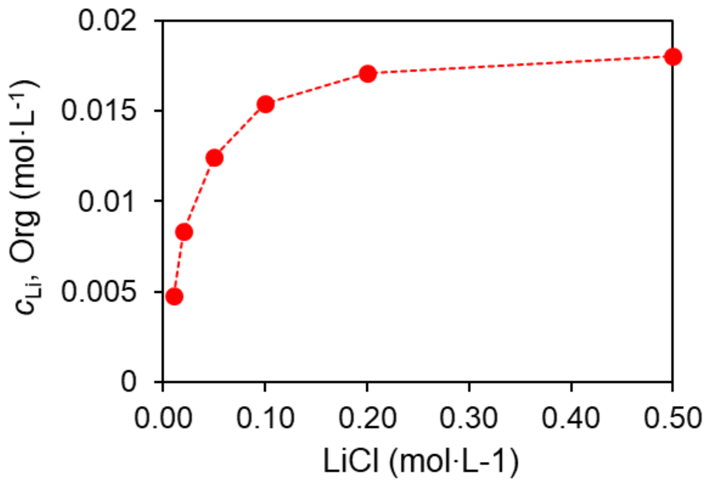

Figure S3. Loading of Li to 0.10 mol·L^{-1} HPMBP and 0.02 mol·L^{-1} Cyanex 923 with various initial LiCl concentrations (horizontal axis). The aqueous solution was buffered at about pH=7.0 using 0.20 mol·L^{-1} sodium dihydrogen phosphate buffer solution. Data for LiCl > 0.50 mol·L^{-1} are not shown because precipitation occurred.
